# Effect of Smear Layer Deproteinization on Gingival Marginal Sealing of Self-Adhesive Flowable Composites: A Micro-CT Study

**DOI:** 10.3390/jfb17090467

**Published:** 2026-09-12

**Authors:** Fulya Aydin, Burcu Öztürk, Gülbike Demirel, Özgür Irmak, Arda Buyuksungur, İsmail Hakki Baltacioğlu, Özgür Karstarli, Kaan Orhan

**Affiliations:** 1 Department of Restorative Dentistry, Faculty of Dentistry, Ankara Medipol University, 06570 Ankara, Türkiye; 2 Department of Restorative Dentistry, Faculty of Dentistry, Ankara University, 06560 Ankara, Türkiye; ozturkbrc93@gmail.com (B.Ö.); gdemirel@ankara.edu.tr (G.D.); o.karstarli@gmail.com (Ö.K.); 3 Department of Restorative Dentistry, Faculty of Dentistry, Cyprus International University, 99258 Nicosia, Türkiye; ozgur.i@icloud.com; 4 Department of Basic Medical Sciences, Faculty of Dentistry, Ankara University, 06560 Ankara, Türkiye; abuyuksungur@ankara.edu.tr; 5 Department of Dentomaxillofacial Radiology, Faculty of Dentistry, Ankara University, 06560 Ankara, Türkiye; knorhan@dentistry.ankara.edu.tr; 6 Department of Oral Diagnostics, Faculty of Dentistry, Semmelweis University, 1088 Budapest, Hungary; 7 Medical Design Application and Research Center (MEDITAM), Ankara University, 06520 Ankara, Türkiye

**Keywords:** adhesive dentistry, deproteinization, hypochlorous acid, restorative dentistry, self-adhesive composites, sodium hypochlorite

## Abstract

Self-adhesive flowable resin composites have been developed to simplify restorative procedures by eliminating separate adhesive application; however, their gingival sealing to dentin remains a concern. This study assessed the gingival marginal sealing of resin composites by measuring silver nitrate solution (AgNO_3_) penetration using micro-computed tomography (µCT). Materials and Methods: Seventy-five human third molars with 150 standardized cavities were allocated into 15 experimental groups based on three surface pretreatments (no pretreatment, sodium hypochlorite solution [NaOCl], and a hypochlorous acid-containing aqueous solution [HCS]) and five restorative materials: three self-adhesive flowable composites (Fusio Self-Adhesive, Vertise Flow, and Nova Compo SF) and two conventional composites (Clearfil Majesty Posterior and Clearfil Majesty Flow). Following thermal cycling, microleakage was quantified by measuring silver nitrate penetration along the gingival floor of the restorations using µCT and statistically analyzed. Results: HCS significantly reduced silver nitrate penetration in all self-adhesive composites (*p* < 0.05), while NaOCl significantly reduced silver nitrate penetration only in the 10-MDP-containing Nova Compo SF (*p* < 0.05). Conventional composites bonded with a self-etch adhesive exhibited significantly lower microleakage than self-adhesive materials (*p* < 0.05). Conclusions: HCS improved the gingival marginal sealing of self-adhesive flowable composites to dentin, whereas the effect of NaOCl was material-dependent. Conventional resin composites used with a self-etch adhesive demonstrated superior gingival marginal sealing compared with self-adhesive flowable composites.

## 1. Introduction

Dental resin composites are the most frequently used esthetic restorative materials in dentistry. These materials are expected to provide long-lasting adhesion to tooth tissues by withstanding the challenging conditions of the oral environment over an extended period [1]. If the marginal adaptation between the tooth structure and restorative material is inadequate, microleakage may occur, potentially resulting in secondary caries, pulpal inflammation, and eventual restoration failure [2].

Due to their chemical nature, resin composites undergo volumetric shrinkage as their monomers convert into polymers [3]. This shrinkage may cause the material to pull away from the tooth surface. Adhesive systems used for bonding resin composites can counteract this phenomenon by limiting separation of the polymerized composite from the tooth tissue [4].

The application of adhesive systems before placement of resin composite involves sequential steps that depend on the adhesive used and requires technical precision for durable adhesion. Manufacturers continually develop adhesive systems that simplify these steps and reduce technique sensitivity [4]. However, even simplified adhesive procedures remain time-consuming and technique-demanding. Consequently, some manufacturers have developed self-adhesive resin composites that eliminate the need for a separate adhesive step. These relatively newer composites can save time and simplify restorative procedures. Functional monomers such as 10-methacryloyloxydecyl dihydrogen phosphate (10-MDP), glycerophosphate dimethacrylate (GPDM), and 4-(2-methacryloyloxy) ethoxycarbonyl phthalic acid (4-META) are added to provide self-adhesion. These monomers modify the “smear layer,” which is created by rotary instruments during tooth preparation, consists of organic and inorganic components, and covers the prepared tooth surface [4,5,6]. While etch-and-rinse adhesives completely remove this smear layer, self-etch adhesive systems, self-etch resin cements, and self-adhesive resin composites modify and incorporate it into the hybrid layer. Notably, self-adhesive resin cements and composites tend to have lower bond strengths than restoratives used with a separate adhesive system [5].

Flowable composites may be used as liners beneath more highly filled restorative composites because their low viscosity facilitates adaptation to cavity surfaces and geometrically complex areas. Self-adhesive flowable composites may further simplify this approach by combining the lining and adhesive steps.

Sodium hypochlorite (NaOCl) solution and hypochlorous acid (HOCl)-containing solutions are both oxidizing deproteinizing agents, but they differ in their chemical characteristics and effects on dentin. Sodium hypochlorite solutions are strongly alkaline and exhibit pronounced proteolytic activity, allowing dissolution of the organic components of the smear layer; however, their action may also affect dentin collagen, and residual oxidizing species may interfere with resin polymerization and bonding. In hypochlorous acid-containing solutions at near-neutral pH, a greater proportion of available chlorine is present as HOCl, which is also capable of removing organic components of the smear layer. These differences in chemical characteristics and their effects on the smear layer and resin–dentin interface have prompted the investigation of both solutions as dentin pretreatment agents [7].

Some researchers have applied deproteinizing solutions such as sodium hypochlorite (NaOCl) or hypochlorous acid solution (HCS) to the smear layer, particularly in dentin and in smear layers containing carious tissue and reported increased bond strengths for self-etch resin cements [8,9,10]. However, the literature lacks sufficient information on the effect of deproteinizing solutions applied before self-adhesive resin composites, indicating a knowledge gap. Therefore, the present study evaluated the effect of NaOCl and HCS as deproteinizing agents on the gingival marginal sealing of self-adhesive flowable resin composites used as thin liners under a conventional restorative composite to dentin. Micro-computed tomography (µCT) was used for its high-resolution, non-destructive, three-dimensional assessment of the tooth–restoration interface. The null hypothesis tested was that the pretreatment with NaOCl and HCS would have no effect on the gingival marginal sealing of self-adhesive flowable resin composites to dentin.

## 2. Materials and Methods

Ethical approval was obtained from the Ethics Committee of Ankara University Faculty of Dentistry (Ref No: 36290600-27). The study design was based on three main groups defined by surface pretreatment and five subgroups defined by the restorative material, resulting in 15 experimental groups. The required sample size of 10 specimens per group was calculated using G*Power 3.1 [11] (α = 0.05; power = 0.80). Seventy-five extracted human third molars were used. All calculus and debris were removed from the tooth surfaces. The extracted teeth were cleaned and disinfected by sequential swabbing with 3% hydrogen peroxide and 2% chlorhexidine [12]. The teeth were then stored in deionized water at 4 °C and used within 3 months.

**Figure 1:**
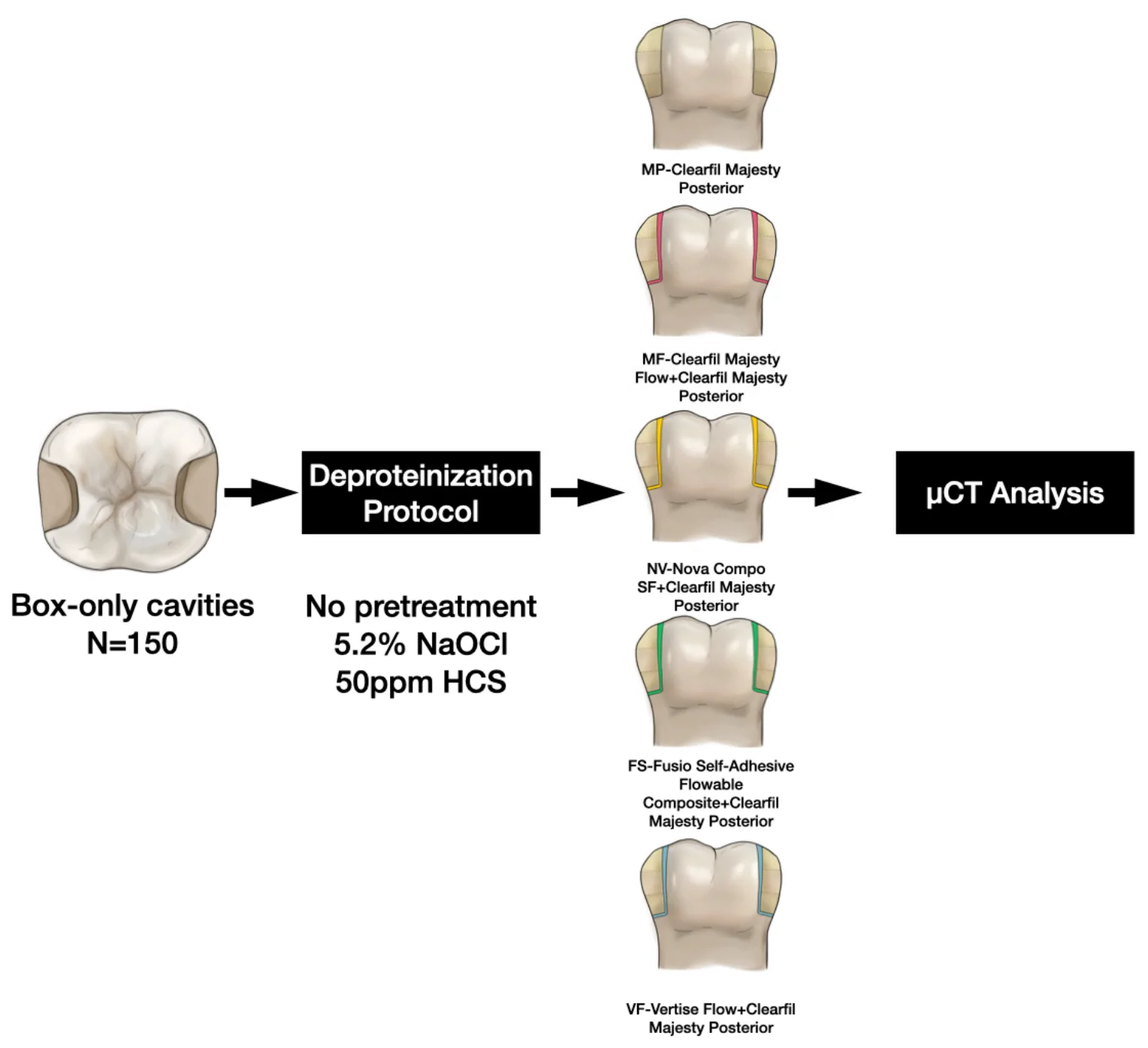
Schematic overview of the experimental protocol and group allocation.

The pretreatment groups were: no pretreatment (control), 5.2% sodium hypochlorite solution (NaOCl; pH 12.6; Chloraxid, Stalowa Wola, Cerkamed, Poland), and a hypochlorous acid-containing aqueous solution (HCS; 50 ppm, pH 7.0; Comfosy, Haccpper Advantec Co., Tokyo, Japan). The latter is produced by neutralizing sodium hypochlorite with hydrochloric acid and diluting the resulting solution in water. The restorative materials were Fusio Self-Adhesive Flowable Composite (FS; Pentron Clinical, Orange, CA, USA), Vertise Flow (VF; Kerr Corporation, Orange, CA, USA), Nova Compo SF (NV; Imicryl, Konya, Türkiye), Clearfil Majesty Posterior (MP; Kuraray Noritake Dental, Tokyo, Japan) as a conventional paste-like composite, and Clearfil Majesty Flow (MF; Kuraray Noritake Dental, Tokyo, Japan) as a conventional flowable composite (Table 1). In each tooth, two standardized “box-only” Class II cavities were prepared, yielding a total of 150 cavities (two per tooth; *n* = 10 per subgroup). Both cavities prepared in the same tooth were assigned to the same experimental condition, including the same deproteinization pretreatment and restorative material. Thus, each experimental subgroup comprised five teeth and ten cavity-level measurements (*n* = 10), with two measurements originating from each tooth. The overall experimental design is presented in Figure 1.

### 2.1. Cavity Preparation

Teeth were cleaned to remove any residual tissue and examined to confirm the absence of caries and cracks. In each tooth, standardized “box-only” cavities were prepared in the mesial and distal aspects. The gingival floor of each cavity was positioned 1 mm below the enamel–cementum junction. Cavity dimensions were standardized to a buccolingual width of 3 mm and a gingival floor width of 2 mm, measured with a Williams periodontal probe. Preparations were performed using diamond cylinder burs (S837G, Hager & Meisinger GmbH, Neuss, Germany) under water cooling; burs were replaced after every five cavities. After preparation, teeth were thoroughly rinsed and gently air-dried.

### 2.2. Deproteinization Protocol

The deproteinizing solution (NaOCl or HCS) was generously applied to the entire dentin surface with a clean microbrush for 15 s, followed by a 30 s water rinse. Following deproteinization, Clearfil DC Activator (Kuraray Noritake Dental, Okayama, Japan), an arylsulfinate salt-containing agent, was used as a reducing agent to counteract the potential residual oxidizing effects of the deproteinizing agents on subsequent adhesive polymerization, as previously described for NaOCl- and HOCl-treated dentin [13].

### 2.3. Restorative Protocol

Prior to the restorative procedures, circumferential metal matrix bands (Adapt SuperCap Matrix No. 2182; Kerr-Hawe, Bioggio, Switzerland) were placed to establish an anatomically correct contour. To prevent gingival overhangs or excess material, the matrices were carefully tightened and burnished against the cavity margins, ensuring close adaptation during composite placement.

For the conventional resin composites (MP and MF), a two-step self-etch adhesive (Clearfil SE BOND 2, Kuraray, Okayama, Japan) was applied and light-cured for 10 s with an LED curing unit (Radii Plus, SDI, Australia; radiant emittance 1500 mW/cm^2^) before placement of the restorative material. In the paste-like conventional composite group (MP), a horizontal incremental technique was used with 2 mm-thick layers, each increment light-cured for 20 s. The cavity was restored with subsequent horizontal increments using the same protocol. In the conventional flowable composite group (MF), a 0.5 mm-thick layer was applied to the cavity floor and side walls and light-cured for 20 s. The remainder of the cavity was then restored with the paste-like composite (MP) using the same horizontal layering technique.

For the self-adhesive flowable composite groups (FS, VF, and NV), a liner layer with a target thickness of approximately 0.5 mm was applied to the cavity floor and walls with a rubbing motion for 20 s and light-cured for 20 s. Before light curing, a clean dental explorer was gently passed through the liner layer to facilitate uniform adaptation and eliminate any entrapped air bubbles. The approximate liner thickness was checked using a periodontal probe, and a visually discernible liner layer was confirmed along the axial line angles before placement of the overlying restorative composite. The cavity was then restored with the paste-like composite (MP) using the same horizontal layering technique, with each increment light-cured for 20 s.

Following restoration, the specimens were finished under water using abrasive discs in a sequence from coarse to extra-fine grit and subsequently stored in distilled water for 24 h. All cavity preparations and restorative procedures were performed by the same operator.

### 2.4. Thermal Aging

After the restorations were completed, specimens underwent 10,000 thermal cycles to simulate approximately 1 year of clinical service [14]. Cycling alternated between water baths at 5 ± 2 °C and 55 ± 2 °C, with a dwell time of 30 s in each bath and a transfer time of 7 s.

### 2.5. Microleakage Analysis with µCT

Following aging, tooth apices were sealed using a self-etch adhesive and a flowable composite. For each tooth, two layers of nail varnish were applied to the entire surface except for a 1 mm band along the gingival tooth–restoration margin. Each group was distinguished by a unique varnish color. Samples were stored in a 50% solution of ammoniacal silver nitrate (AgNO_3_) prepared according to the previously described silver-tracer protocol [15] in darkness for 12 h. The 50 wt% ammoniacal silver nitrate solution prepared according to this protocol has been reported to have a pH of 9.5 [15]; however, the pH of the solution used in the present study was not independently measured. Samples were then rinsed with water for 2 min, and then immersed in a photo-developing solution (GBX Developer, Carestream Dental, Atlanta, GA, USA) prepared according to the manufacturer’s instructions, for 8 h under illumination [15]. The pH of the working developing solution was not independently measured. After immersion, samples were brushed under running water.

Microleakage was analyzed using a high-resolution µCT scanner (Bruker SkyScan 1275; Bruker, Kontich, Belgium). The scanning protocol used a 1 mm Al filter at 80 kVp and 125 µA with a rotation increment of 0.2° using 30 µm/pixel. Sensor air calibration was performed before each session for flat-field correction. Each sample was scanned with a 360° rotation. Reconstruction parameters, ring artifact correction was 7, Beam Hardening Correction was 38%, and smoothing was 3. Image Conversion limits were set to 0–0.16.

**Figure 2:**
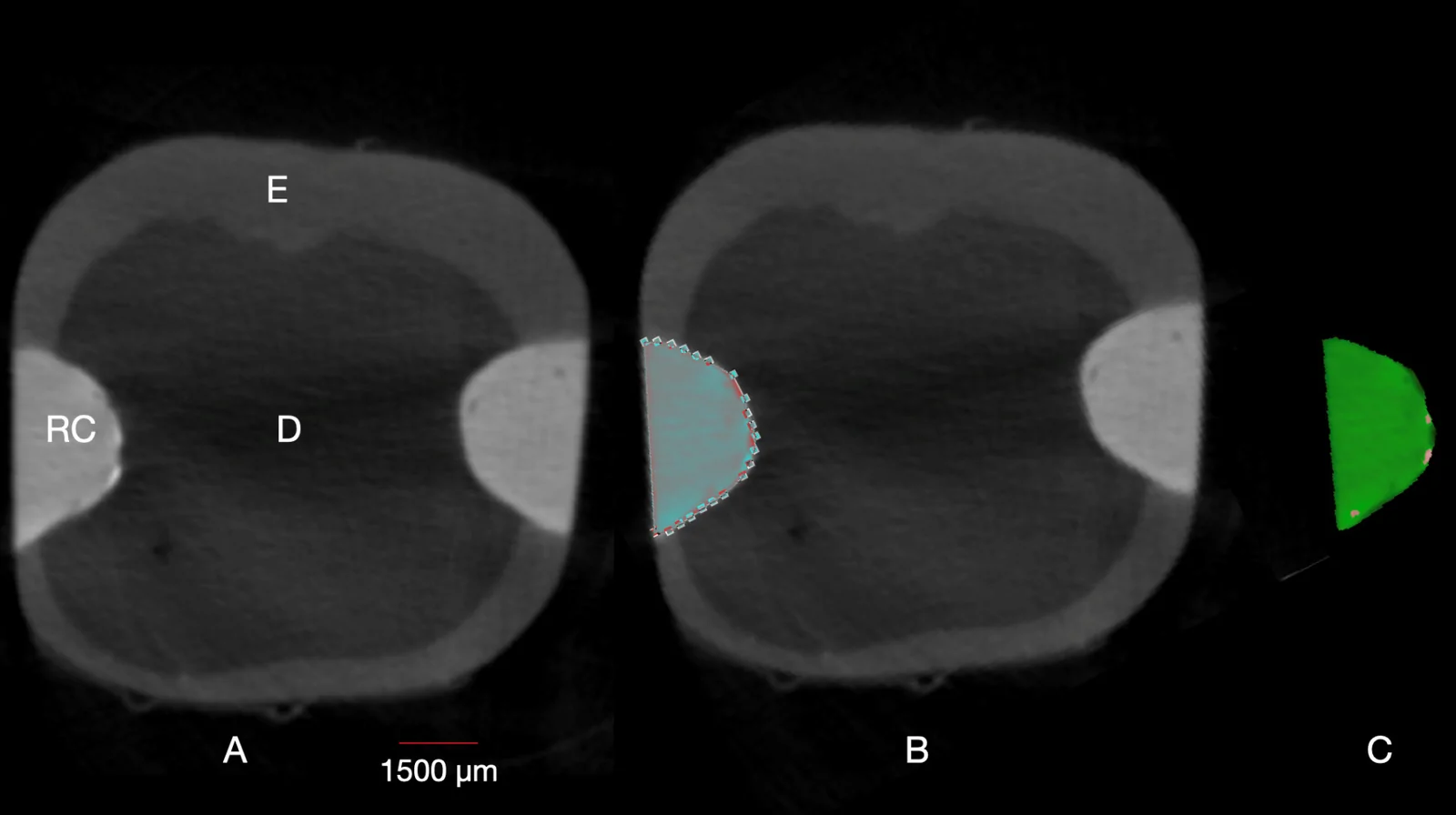
Reconstructed µCT scan in the horizontal section. (**A**): Enamel, dentin, and composite resin structures. (**B**): Defined region of interest (ROI) (blue and red) encompassing the cavity margins. (**C**): Within the ROI, AgNO_3_ (pink) and composite resin (green) are displayed; enamel and dentin have been excluded from the image. Abbreviations: D, dentin; E, enamel; RC, resin composite.

Image acquisition and analysis were performed by a second operator blinded to the group allocation. The resulting 2D images were reconstructed into 3D models and used to quantify the volume of silver nitrate penetration for each restoration (Figure 2). The extent of silver nitrate penetration at the gingival interface was quantified using CTAn software (version 1.23.0.2, Bruker Micro-CT, Kontich, Belgium), CTVox software (version 1.23.0.2, Bruker Micro-CT, Kontich, Belgium) was used to 3D rendering of the samples. The total restoration volume was used as a normalization factor to account for minor variations in restoration size among specimens; it did not define the region in which silver nitrate penetration was evaluated. Microleakage (%) was calculated as the ratio of the volume of silver nitrate penetration at the gingival floor to the total restoration volume, multiplied by 100.

$$\mathrm{Microleakage}\;(\%)=\left(\frac{\mathrm{Volume}\;\mathrm{of}\;\mathrm{silver}\;\mathrm{nitrate}\;\mathrm{penetration}\;\mathrm{at}\;\mathrm{the}\;\mathrm{gingival}\;\mathrm{floor}}{\mathrm{Total}\;\mathrm{restoration}\;\mathrm{volume}}\right)\times 100$$

### 2.6. Statistical Analysis

Normality was assessed based on the conditional residuals of the fitted model using the Shapiro–Wilk test and graphical inspection of the Q–Q plot. Homogeneity of conditional residual variances across experimental groups was evaluated using the Brown–Forsythe test. The effects of deproteinization pretreatment, restorative composite, and their interaction on silver nitrate penetration were analyzed using a linear mixed-effects model, with pretreatment and composite included as fixed effects and tooth included as a random intercept to account for the correlation between the two cavity-level measurements obtained from the same tooth. Fixed effects were evaluated using Type III tests with Satterthwaite’s approximation for denominator degrees of freedom. In the presence of a significant pretreatment × composite interaction, estimated marginal means were used to perform simple-effects pairwise comparisons, with Tukey adjustment for multiple comparisons. The significance level was set at α = 0.05. All statistical analyses were performed using R (version 4.5.2; R Foundation for Statistical Computing, Vienna, Austria) in RStudio (version 2026.08.0+187; Posit Software, PBC).

## 3. Results

**Figure 3:**
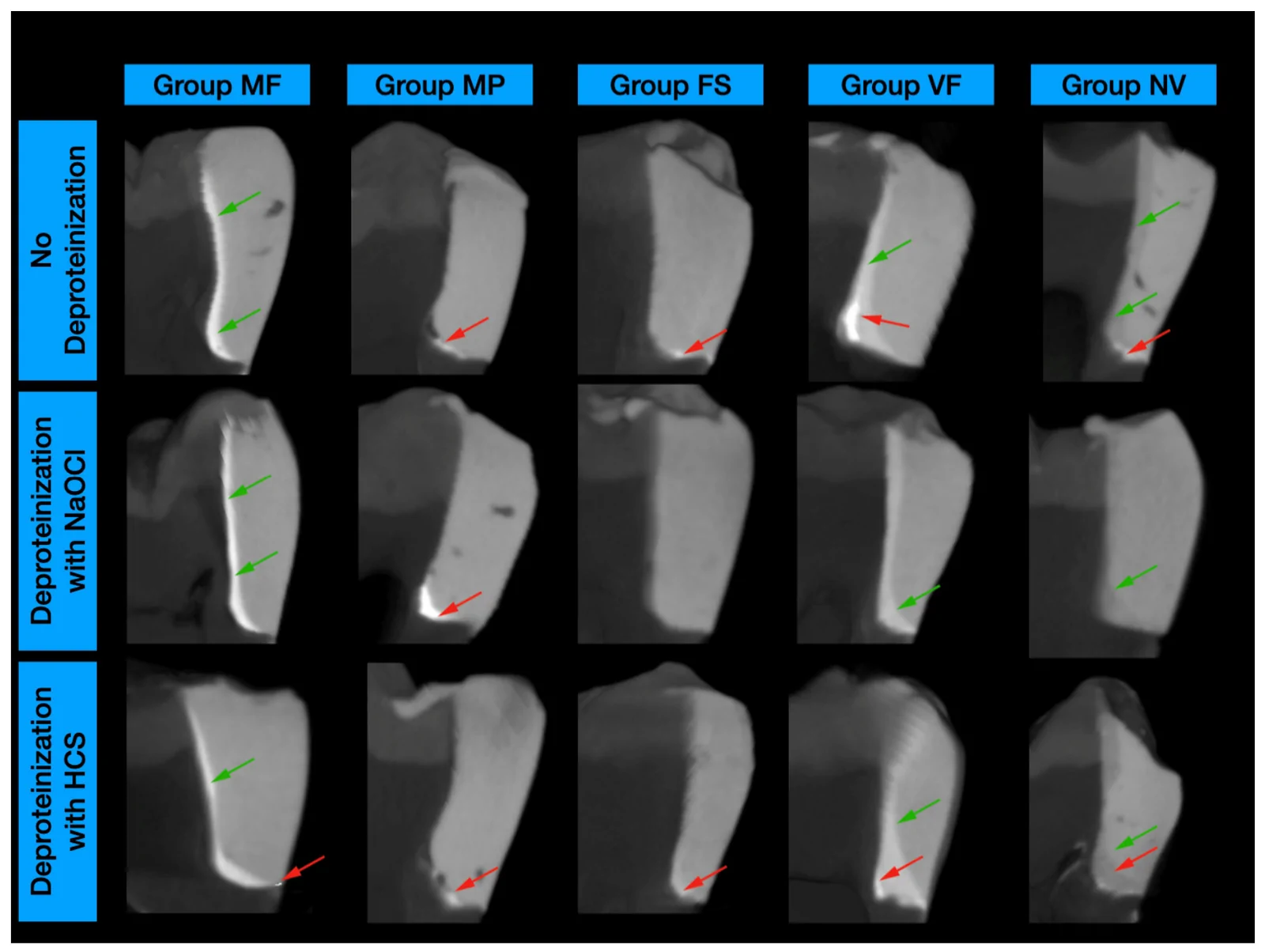
Representative µCT reconstructions of cavities restored with different self-adhesive composite resins. Green arrows indicate the interfacial liner composite layer, while red arrows highlight silver nitrate (AgNO_3_) infiltration originating from the restoration margins.

Conditional residuals showed no significant departure from normality (Shapiro–Wilk W = 0.985, *p* = 0.106). The Brown–Forsythe test indicated no significant heterogeneity of residual variance across the fixed-effect cells (F(14, 135) = 1.138, *p* = 0.331). The linear mixed-effects model demonstrated a significant main effect of deproteinization pretreatment (F(2, 60) = 47.92, *p* < 0.001), a significant main effect of composite type (F(4, 60) = 41.98, *p* < 0.001), and a significant pretreatment × composite interaction (F(8, 60) = 18.62, *p* < 0.001), indicating that the effect of pretreatment depended on the restorative composite. The mean (±SD) leakage values and multiple comparisons are presented in Table 2. Representative micro-CT images are shown in Figure 3.

In the control groups (no deproteinization), the conventional resin composites (MF and MP) showed lower AgNO_3_ penetration than the self-adhesive flowable materials (NV, FS, VF) (*p* < 0.05). There were no statistically significant differences either between the two control materials themselves or among the self-adhesive materials (*p* > 0.05). In the HCS groups, MF showed significantly lower AgNO_3_ penetration than FS and VF (*p* < 0.01), and both MF and MP showed significantly lower penetration than NV (*p* < 0.001); the remaining pairwise comparisons were not statistically significant (*p* > 0.05). For NaOCl groups, MF and NV exhibited the lowest levels of silver nitrate penetration; both differed significantly from the other materials (*p* < 0.05).

For each self-adhesive material, HCS deproteinization produced a statistically significant reduction in silver nitrate penetration compared with the control (*p* < 0.05). In contrast, NaOCl significantly decreased penetration in both the NV and FS groups compared with the control (*p* < 0.05), although the reduction in FS was marginally significant (*p* = 0.049); no significant improvement was observed in VF. When the two agents were compared, HCS resulted in lower penetration than NaOCl in the FS and VF groups (*p* < 0.05), whereas for NV, NaOCl produced lower penetration than HCS (*p* < 0.05).

## 4. Discussion

This study evaluated the influence of NaOCl and HCS dentin deproteinization on the gingival marginal sealing of different restorative composites. The null hypothesis was partially rejected, as HCS significantly reduced silver nitrate penetration in self-adhesive composites compared to the control, whereas NaOCl produced significant reductions in NV and FS, with the reduction in FS being marginally significant.

The use of flowable composites as liners may influence the behavior of the overlying restorative composite. In Class I restorations, the presence and thickness of a flowable liner were shown to affect polymerization shrinkage vectors, with the absence of a liner resulting in greater shrinkage vectors [16]. In Class II restorations, the restorative application method also influenced shrinkage behavior, and the use of a thin flowable liner reduced shrinkage vectors [17]. These findings support the potential benefit of flowable liners in modifying polymerization shrinkage behavior. However, unlike these studies, which evaluated polymerization shrinkage vectors and volumetric shrinkage, the present study assessed silver nitrate penetration at the gingival interface. Therefore, their findings should be considered complementary rather than directly comparable to the microleakage outcomes of the present study.

The present analysis was limited to the gingival floor because this region represents the most clinically critical site for marginal sealing and microleakage in Class II restorations. Unlike enamel margins, gingival margins are frequently located in dentin or cementum, where bonding is inherently more challenging and interfacial leakage is more likely. Consequently, evaluating this region provides a clinically relevant assessment of the interfacial sealing performance of restorative materials and surface pretreatments.

Marginal integrity can be evaluated using different imaging methods, including SEM, TEM, and µCT. SEM provides high-resolution morphological assessment of restoration margins but is limited to the exposed surface or section examined and does not provide volumetric information. TEM provides substantially higher-resolution characterization of the adhesive interface and ultrastructural features, although it requires extensive specimen preparation and is inherently limited to selected ultrathin sections. In contrast, µCT enables non-destructive, three-dimensional evaluation of the tooth–restoration interface and volumetric assessment of tracer penetration. Therefore, µCT was used in the present study to quantify silver nitrate penetration at the gingival interface. Previous micro-CT studies have demonstrated the applicability of silver nitrate as a radiopaque tracer for three-dimensional assessment of marginal leakage in composite restorations. In Class II restorations, greater tracer penetration has been reported at dentin/cementum margins than at enamel margins, supporting the particular relevance of the gingival interface for leakage assessment [18]. In addition, volumetric micro-CT approaches based on silver nitrate penetration and image segmentation have demonstrated that interfacial leakage can be quantified three-dimensionally and may exhibit non-uniform penetration patterns that are not fully represented by selected two-dimensional sections [19].

Dentin deproteinization aims to reduce the organic content of the smear layer and superficial dentin by partially removing exposed collagen fibrils rather than completely eliminating the collagen matrix [7,13,20,21,22]. Previous morphological studies have reported that partial deproteinization can produce a relatively mineral-rich superficial dentin substrate, with increased exposure of dentinal tubules and changes in resin infiltration depending on the extent of collagen removal and the treatment protocol employed [7,20,21,22]. Deproteinization has also been associated in previous microscopic studies with the formation of a “reverse hybrid layer”, a resin–mineral interdiffusion zone structurally distinct from the conventional resin-infiltrated collagen hybrid layer [23,24].

Among the solutions proposed for dentin deproteinization, NaOCl and HCS are among the most widely investigated [9,13,21,22,25,26,27,28,29]. NaOCl, known for its strong proteolytic activity, effectively removes organic material and produces a porous and irregular dentin [27] that favors resin infiltration and resin tag formation [24]. The strongly alkaline nature of NaOCl also contributes to its antimicrobial activity. In contrast to the strongly alkaline NaOCl solution, HCS is an acidified sodium hypochlorite-based aqueous solution in which available chlorine is present as HOCl and OCl^−^ in pH-dependent proportions. At its near-neutral pH, a greater proportion of available chlorine is present as HOCl. HCS exerts a similar deproteinizing effect and its near-neutral pH and chlorine specification allow effective smear layer deproteinization at lower chlorine concentrations [7,13,26,29]. However, near-neutral HCS does not provide the antimicrobial contribution associated with the strongly alkaline pH of conventional NaOCl. Furthermore, HCS can be more easily rinsed away from the treated surface, thereby reducing the detrimental impact on bonding often associated with residual NaOCl [13,30]. Thus, the potential advantages of HCS discussed here are limited to its use as a dentin deproteinizing pretreatment and should not be interpreted as indicating overall superiority to NaOCl.

Despite these advantages, no consensus has been reached regarding the clinical use of deproteinizing agents. Some studies have reported improved marginal adaptation with NaOCl [31] whereas others favored HCS [7,28]. A recent meta-analysis concluded that neither pretreatment consistently enhances the bonding effectiveness of self-etch adhesives to dentin [25]. Furthermore, optimal concentration, application time, or rinsing protocol remain unclear, although many studies have employed 15 s application with 6% NaOCl or 50 ppm HCS and 30 s rinsing [7,26,30,32,33,34]. To ensure comparability with previous work, the same standardized protocol was used in this study.

Residual oxidizing activity of NaOCl and HCS represents another concern, as it may inhibit resin polymerization. Application of reducing agents or antioxidants, such as sodium ascorbate, rosmarinic acid, or sodium p-toluene-sulfonate, has been shown to counteract this effect by neutralizing the oxidized substrate surface and facilitating adhesive polymerization [26,35,36,37,38]. In the present study, Clearfil Dual Cure Activator, an arylsulfinate-containing agent, was used as a reducing agent following deproteinization. Arylsulfinate-containing agents have been used for this purpose after oxidative dentin pretreatment. However, the role of Clearfil DC Activator may not be limited to the reduction of residual oxidizing species. The arylsulfinate component can also participate in polymerization initiation, and recent evidence has shown that its application after oxidative dentin treatment can improve both adhesive degree of conversion and bond strength [39]. Thus, the potential contribution of Clearfil DC Activator to adhesive polymerization should also be considered when interpreting the effects of the deproteinization protocols.

In the present study, HCS significantly reduced microleakage in all self-adhesive composites tested, whereas NaOCl significantly reduced microleakage in NV and FS, although the effect in FS was marginally significant. These findings should be interpreted in light of the mechanism of smear layer deproteinization. Although deproteinizing agents are intended to reduce the organic component of the smear layer, the protocols used in the present study do not produce complete deproteinization. Sanon et al. [13] demonstrated that application protocols comparable to those used in the present study resulted in approximately 33% and 44% deproteinization following NaOCl and HCS treatment, respectively. Furthermore, SEM analysis showed that the smear layer remained present after both treatments, indicating that deproteinization occurred primarily within the organic component of the smear layer while its inorganic phase persisted. Accordingly, the improved gingival marginal sealing observed after HCS pretreatment should be interpreted as the result of more effective partial smear layer modification rather than complete collagen removal. Previous ultrastructural observations have nevertheless shown that such partial deproteinization can substantially modify the adhesive interface. Thanatvarakorn et al. [7] demonstrated thinning or elimination of the hybridized smear layer at the resin–dentin interface following NaOCl or HCS pretreatment, with HCS producing more consistent elimination after clinically relevant application times and exhibiting less nanoleakage than NaOCl. These findings indicate that partial smear layer modification is sufficient to alter the morphology of the adhesive interface, which may contribute to improved interfacial sealing, even without complete removal of the organic matrix. Both 15- and 30-s applications significantly reduced the organic component of the smear layer; however, TEM observations showed that a thin hybridized smear layer remained after 15-s NaOCl treatment and was completely eliminated after 30 s, whereas HCS completely eliminated the hybridized smear layer after both application times.

A significant interaction between deproteinization protocol and composite type was observed, indicating that the effects of HCS and NaOCl are material-dependent. This emphasizes the importance of considering the chemical composition of restorative materials when selecting a deproteinizing pretreatment. HCS produced consistent improvements across all self-adhesive composites although the mechanism underlying this response cannot be determined from the present microleakage data. In contrast, the material-dependent response observed after NaOCl pretreatment may reflect differences in material composition and their response to the pretreatment protocol. FS contains 4-META, VF incorporates GPDM, whereas NV combines both 10-MDP and 4-MET. Although these differences in functional monomer composition may contribute to material-dependent behavior, the present study did not directly assess monomer–mineral interactions or interfacial chemistry. Therefore, the responses of NV and FS cannot be attributed to specific functional monomers or monomer–hydroxyapatite interactions based on the present data. Potential interactions or competition among functional monomers, including the 10-MDP and 4-MET present together in NV, cannot be determined from this study either.

Finally, it should be emphasized that microleakage studies, including μCT-based quantitative assessments, provide valuable information on interfacial sealing but cannot fully predict clinical performance. The μCT acquisition used for volumetric assessment of silver nitrate penetration does not provide the resolution required to characterize fine interfacial morphology or individual tracer pathways. Accordingly, the observed microleakage patterns cannot be directly correlated with dentin surface characteristics or interfacial ultrastructure. The discussion of possible effects of deproteinization on the dentin substrate is therefore based on findings from previous studies rather than direct morphological observations in the present study. Complementary microscopic examination of sectioned specimens was not performed, and future studies combining μCT with microscopic evaluation would be useful to correlate volumetric leakage with interfacial morphology. This study also has several limitations. Although the staining and developing protocol was applied consistently across all experimental groups, the possibility that prolonged exposure to these solutions may have affected the restorative interfaces cannot be completely excluded. No long-term aging was performed, which may influence the durability of the restorations. Only a single concentration and application time for each deproteinizing pretreatment was tested, which may not fully reflect the variability encountered in clinical practice. In addition, Clearfil DC Activator was applied only after deproteinization and was not used in the untreated control groups. This was intended to preserve the control condition as the baseline restorative protocol without deproteinization or subsequent reducing treatment. However, because the activator may contribute not only to the reduction of residual oxidizing species but also to adhesive polymerization, its independent contribution cannot be separated from that of the preceding deproteinization treatment in the present experimental design. Accordingly, the findings should be interpreted as reflecting the effects of the complete deproteinization/sulfinate pretreatment protocols rather than the isolated chemical effects of NaOCl or HCS alone. Future studies incorporating corresponding groups with and without the activator would be required to distinguish the independent and interactive effects of the deproteinizing agents and Clearfil DC Activator. Despite these limitations, the marked reduction in silver nitrate penetration following HCS pretreatment suggests a potential for improved longevity of adhesive restorations, particularly when viscous self-adhesive composites are used.

## 5. Conclusions

Within the limits of this study, HCS provided more favorable outcomes for self-adhesive flowable composites than NaOCl, as demonstrated by reduced silver nitrate penetration at the gingival margin. NaOCl showed a more material-dependent effect, with significant reductions in silver nitrate penetration observed in both NV and FS, although the effect was more pronounced in NV. These material-dependent differences highlight that the choice of deproteinizing pretreatment should be guided by the composition of the restorative material.

## Figures and Tables

**Table 1:** The restorative materials and adhesive system used in the study.

Material Type	Brand Name (Abbreviation)	Organic Contents	Inorganic Contents	Manufacturer
Adhesive System	Clearfil SE Bond	Primer: MDP, HEMA, Hydrophilic dimethacrylate, Water. Adhesive: MDP, Bis-GMA, HEMA, Hydrophobic dimethacrylate, dl- Camphorquinone, N, N-Diethanol-p- toluidine, Silanated colloidal silica	Silanated colloidal silica	Kuraray, Okayama, Japan
Nano-hybrid composite	Clearfıl Majesty Flow (MF)	TEGDMA, hydrophobic aromatic dimethacrylate, camphorquinone, accelerators, initiators, pigments.	Silanated barium glass filler, silanated silica filler	Kuraray Co., Ltd., Osaka, Japan
Nano-hybrid composite	Clearfil Majesty Posterior (MP)	BİS-GMA, hydrophobic aromatic dimethacrylate, camphorquinone,	Silanated glass ceramics; surface-treated alumina microfiller; silanated silica filler	Kuraray Co., Ltd., Osaka, Japan
Self-Adhesive Composite	Fusio Liquid Dentin (FS)	UDMA, TEGDMA, HEMA, 4-MET	Amorphous silica nanosized, silanized Barium glass	Pentron Clinical, Orange, CA, USA
Self-Adhesive Composite	Vertise Flow (VF)	GPDM, UDMA, BİS-GMA, HEMA, photoinitiator, methacrylate comonomers,	Prepolymerized filler containing barium glass filler, nano-sized ytterbium fluoride, nano-sized colloidal silica	Kerr, Orange, CA, USA
Self-Adhesive Composite	Nova Compo SF (NV)	BİS-GMA, 10-MDP, 4-META, ULS monomer, dimetakrilat, initiators and stabilizer	Silicate filler, Floro alümina	Imicryl, Konya, Turkey

Abbreviations: Bis-GMA: bisphenol A-glycidyl methacrylate, TEGDMA: Triethylene glycol dimethacrylate; ULS: Ultra Low Shrinkage, 4-META: 4-methacryloyloxyethy trimellitate anhydride, 10-MDP: 10-methacryloyloxy-decyl-dihydrogen-phosphate; UDMA: Urethane-dimethacrylate; HEMA: Hydroxyethyl Methacrylate; 4-MET: 4-methacryloxyethyl trimellitic acid; GPDM: glycero-phosphate dimethacrylate.

**Table 2:** Mean microleakage (%) (±SD) for all restorative materials according to deproteinizing agent. (MF, Clearfil Majesty Flow; MP, Clearfil Majesty Posterior; NV, Nova Compo SF; FS, Fusio Self-Adhesive Flowable Composite; VF, Vertise Flow).

	Conventional Composites		Self-Adhesive Composites		
	MF	MP	NV	FS	VF
No agent (Control)	1.85 ± 0.30 ^Aa^	1.85 ± 0.35 ^Aa^	3.22 ± 0.40 ^Ba^	3.15 ± 0.33 ^Ba^	3.16 ± 0.37 ^Ba^
NaOCl	1.73 ± 0.33 ^Aa^	2.50 ± 0.49 ^Bb^	1.39 ± 0.29 ^Ab^	2.75 ± 0.52 ^Ba^	2.86 ± 0.21 ^Ba^
HCS	1.40 ± 0.23 ^Aa^	1.64 ± 0.34 ^Aa^	2.40 ± 0.53 ^Bc^	2.09 ± 0.29 ^Bb^	2.05 ± 0.25 ^Bb^

Means sharing a superscript letter are not significantly different (*p* > 0.05). Uppercase letters compare means in each row. Lowercase letters compare means in each column.

## Data Availability

The original contributions presented in the study are included in the article, further inquiries can be directed to the corresponding author.
